# Synthesis, characterization and toxicological evaluation of maltodextrin capped cadmium sulfide nanoparticles in human cell lines and chicken embryos

**DOI:** 10.1186/1477-3155-10-47

**Published:** 2012-12-27

**Authors:** Patricia Rodríguez-Fragoso, Jorge Reyes-Esparza, Angel León-Buitimea, Lourdes Rodríguez-Fragoso

**Affiliations:** 1 Departamento de Fisica, CINVESTAV - I.P.N Apartado, Postal 14-740, Mexico, 07000, Mexico; 2 Facultad de Farmacia, Universidad Autónoma del Estado de Morelos Cuernavaca, Mexico, 62210, Mexico

**Keywords:** Semiconductor quantum dot, Nanoparticles, Cadmiun sulfide, Cytotoxicity, Cell proliferation, Oxidative stress, Radical oxygen species, Apoptosis, Necrosis, Embriotoxicity

## Abstract

**Background:**

Semiconductor Quantum dots (QDs) have become quite popular thanks to their properties and wide use in biological and biomedical studies. However, these same properties entail new challenges in understanding, predicting, and managing potential adverse health effects following exposure. Cadmium and selenium, which are the major components of the majority of quantum dots, are known to be acutely and chronically toxic to cells and organisms. Protecting the core of nanoparticles can, to some degree, control the toxicity related to cadmium and selenium leakage.

**Results:**

This study successfully synthesized and characterized maltodextrin coated cadmium sulfide semiconductor nanoparticles. The results show that CdS-MD nanoparticles are cytotoxic and embryotoxic. CdS-MD nanoparticles in low concentrations (4.92 and 6.56 nM) lightly increased the number of HepG2 cell. A reduction in MDA-MB-231 cells was observed with concentrations higher than 4.92 nM in a dose response manner, while Caco-2 cells showed an important increase starting at 1.64 nM. CdS-MD nanoparticles induced cell death by apoptosis and necrosis in MDA-MD-231 cells starting at 8.20 nM concentrations in a dose response manner. The exposure of these cells to 11.48-14.76 nM of CdS-MD nanoparticles induced ROS production. The analysis of cell proliferation in MDA-MB-231 showed different effects. Low concentrations (1.64 nM) increased cell proliferation (6%) at 7 days (p < 0.05). However, higher concentrations (>4.92 nM) increased cell proliferation in a dose response manner (15-30%) at 7 days. Exposures of chicken embryos to CdS-MD nanoparticles resulted in a dose-dependent increase in anomalies that, starting at 9.84 nM, centered on the heart, central nervous system, placodes, neural tube and somites. No toxic alterations were observed with concentrations of < 3.28 nM, neither in cells nor chicken embryos.

**Conclusions:**

Our results indicate that CdS-MD nanoparticles induce cell death and alter cell proliferation in human cell lines at concentrations higher than 4.92 nM. We also demonstrated that they are embryotoxic. However, no toxic effects were observed with doses lower than 3.28 nM in neither cells nor chicken embryos. The CdS-MD nanoparticles used in this study can be potentially used in bio-imaging applications. However, further studies using mammalian species are required in order to discard more toxic effects.

## Background

The emergence of quantum dots (QDs) as biological imaging agents has been quick due to the extremely favorable optical properties associated with high quality quantum scale semiconducting materials. Most QDs are made of heavy metal ions (e.g., Cd^++^), which may result in potential in vitro toxicity that hampers their practical applications [1-3]. Advances in synthetic and surface ligand chemistry have provided materials with an almost unrivalled photostability in aqueous solution.

Cadmium selenide or cadmium telluride particles are considered the most suitable emitting ‘core’ materials because of their bright emission in the visible range and near the infrared region of the electromagnetic spectrum [4-6]. However, problems such as the unsuitability of the capping agents, the retention of particles over a certain size, biological magnification, and specifically, the breakdown and decomposition products of these inorganic materials have been suggested.

Recently, polymers that can act as coordination sites for cadmium ion aggregation have protected semiconductor nanoparticles. CdS nanoparticles protected with starch and, in particular, amylose, form a wide range of inclusion complexes for numerous guest molecules [7]. Soluble starch added during the synthesis has been used as a capping agent in the synthesis of CdS and CdSe nanoparticles, resulting in well-controlled and uniform particles sizes of cadmium-rich nanoparticles [8].

Systematic cytotoxicity assessment of QDs is of critical importance given their potential biological and biomedical applications [9,10], and a large amount of studies on the cytotoxicity of QDs have been carried out for this purpose [11-13]. Different cellular lines and different sized quantum dots with various coatings have been used, which makes it very difficult to predict whether a cell would experience negative effects when exposed to quantum dots. Cadmium and selenium, which are the major components of the majority of quantum dots, are known to be acutely and chronically toxic to cells and organisms [14-16]. Protecting the core can, to some degree, control toxicity related to cadmium and selenium leakage. However, the change in the physicochemical and structural properties of engineered quantum dots could be responsible for a number of material interactions that could also have toxicological effects.

Here we will synthesize Cadmium sulfide semiconductor nanoparticles and coat them with maltodextrin polymer (CdS-MD) in order to give rise to a dispersed crystalline structure with a particle size in the range of 3nm. Maltodextrin contain linear amylose and branched amylopectin degradation products from the enzymatic hydrolysis of starches [17]. They represent a mixture of saccharides with a broad molecular weight distribution, depending on the dextrose equivalent (DE), which reflects the degree of hydrolysis. Higher DE leads to a decrease in average molecular weight and a change in physicochemical properties. Maltodextrin is a polysaccharide with high encapsulation activity [18]. Maltodextrin has been previously used as a carrier in the proniosome preparation, allowing for flexibility in the amounts of surfactant and other components [19]. After surface coating, we carried out the characterization and toxicological evaluation of CdS-MD nanoparticles protected with maltodextrin in human cell lines and chicken embryos.

## Results

### Synthesis and characterization of CdS-MD nanoparticles

**Figure 1:**
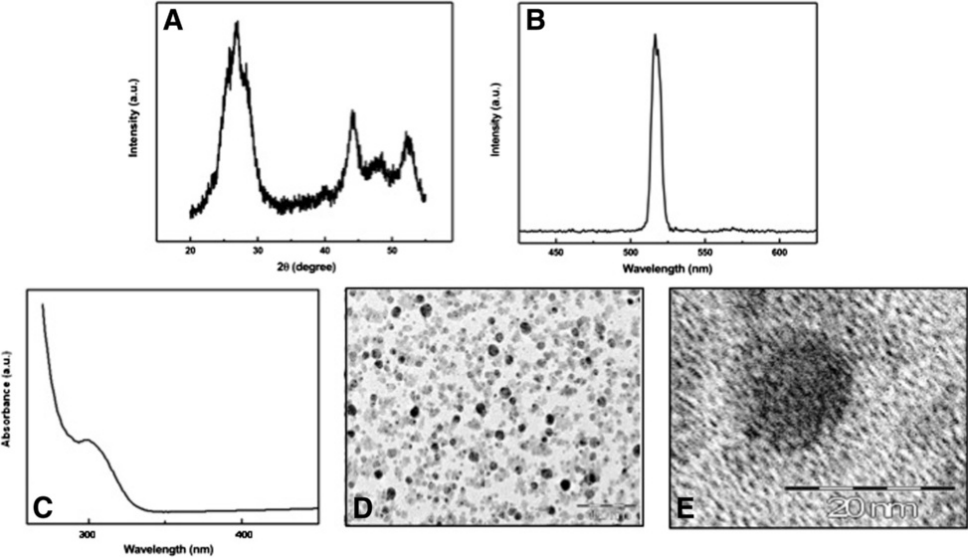
**Quantum dot particles’ formation and characterization. **(**A**) X-ray diffraction patterns of CdS-MD nanoparticles. (**B**) Emission profiles of CdS-MD nanoparticles. (**C**) UV-visible spectrum of CdS-MD nanoparticles. (**D** and **E**) TEM images of CdS-MD nanoparticles. The particles appear evenly spread in the polymeric matrix of maltodextrin. Although some clusters of two to four QDs are visible, most of the QDs are isolated, suggesting that the majority are monodispersed single QD.

Figure 1A shows the XRD pattern of a typical CdS-MD nanoparticles sample. The XRD peaks are very broad, indicating the very fine size of the sample grains. The XRD pattern exhibits prominent broad peaks at 2*θ* values of 26.5°, 43.96° and 52.13°, which could be indexed as scattering from the (111), (220) and (311) cubic phase CdS planes, respectively and according to JCPDS file no. 10–454. By using the Scherrer´s equation *d = 0.8λ/βcosθ*, where *λ* is the wavelength of the X-ray radiation, *β* is the full width at half maximum (FWHM) of the (111) peak, and *θ* is the angle of diffraction, the average size of the CdS-MD nanoparticles was determined to be of the order of 3 nm.

The CdS-MD nanoparticles emission spectrum is shown in Figure 1B. The spectrum exhibits a strong band at 520cm^-1^, and show narrower and more symmetric emission spectra in comparison with organic dyes and fluorescent proteins. The morphology and size of the CdS-MD nanoparticles were observed by TEM. The TEM image in Figure 1D shows a sphere-shaped nanoparticle forming nanoclusters and typical crystalline planes of CdS-MD. Figure 1E shows a close-up of the CdS-MD nanoparticles. These results illustrate the synthesis of CdS-MD nanoparticles through the reduction of Cd^+^ inside the nanoscopic maltodextrin structure.

The CdS-MD nanoparticles concentration was determined from the UV–vis spectrum (Figure 1C), using the Beer-Lambert law:

(1) A=ε∗C∗L

where *A, ε, C* and *L* are absorbance of the excitonic peak, molar extinction coefficient (L mol^-1^ cm^-1^), CdS-MD nanoparticles concentration (mol L^-1^), and path length of the cuvette in which the sample is contained (cm), respectively. The size of CdS-MD nanoparticles is directly related to the excitonic peak in the UV–vis absorption spectrum and also, the molar extinction coefficient *ε* depends on the size one. For determining the molar extinction coefficient *ε*, empirical functions correlating the size of CdS with the position of the first excitonic peak (*λ*) in their UV–vis absorption spectrum and with the molar extinction coefficient as given by Eqs. 2, 3 [20].

(2) $$\begin{array}{l} R=\left(-6.6521x10^{-8}\right)\lambda^{3}\\ \;+\left(1.9557x10^{-4}\right)\lambda^{2}–\left(9.2352x10^{-2}\right)\lambda \\ \;+\left(13.29\right) \end{array}$$

(3) $$\varepsilon =21536\;{\left(R\right)}^{2.3}$$

From the above equations, the estimated radius of the CdS-MD is about 1.5 nm, which is of the same order obtained from the X-ray spectrum, the size of the cuvette *L* was fixed at 1 cm. The concentration of 1μg/ml CdS-MD nanoparticles from the Eq. 1 is of the order of 1.64 nM.

### Effect of CdS-MD nanoparticles on cell viability

**Figure 2:**
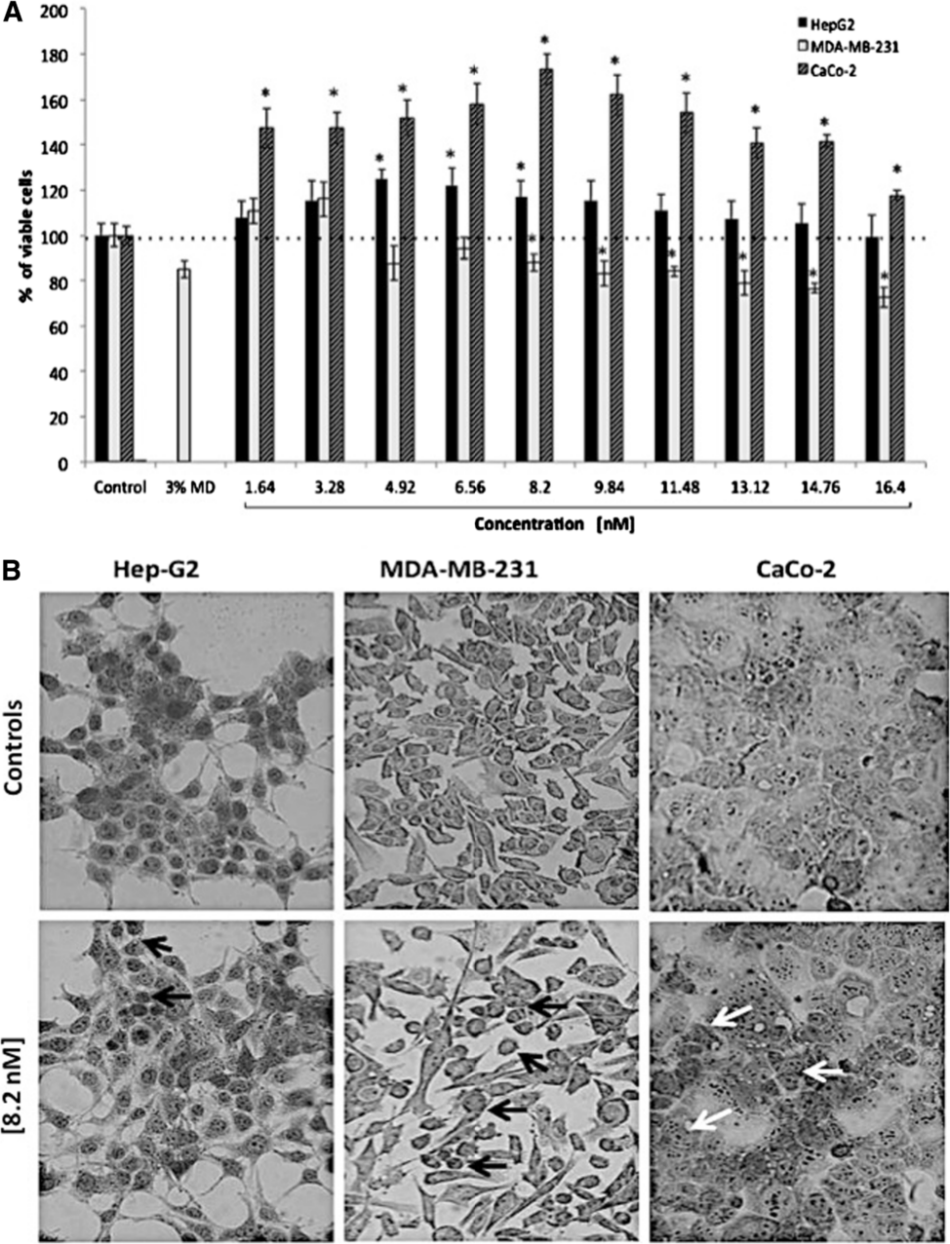
**Effect of CdS-MD nanoparticles on cell viability of human cell lines. **(**A**) Effect of CdS-MD nanoparticles on cell viability on HepG2, MDA-MB-231, and CaCo-2 cells. Cells were exposed in cultured medium with different concentrations of CdS-MD nanoparticles for 24 h. Results are expressed as percentage of cell viability as compared to control group. Data are presented as the mean ± SD of at least three independent experiments. *p <0.05 as compared with control group. (**B**) Morphological analysis of each cell line treated with 8.20 nM of CdS-MD nanoparticles; black arrows indicate apoptotic cells and white arrows indicate cells in division (40X).

Figure 2A shows the effect of CdS-MD nanoparticles on cell viability in human cell lines. As we can see, CdS-MD nanoparticles increased the number of hepatic cells (HepG2) in a 22, 20 and 18% with concentrations of 4.92, 6.56, 8.20 nM, respectively (p < 0.05). Breast cells (MDA-MB-231), on the other hand, showed a significant reduction in the number of viable cells at concentrations higher than 6.56 nM in a dose dependent manner (p < 0.05). Intestinal cells (CaCo-2) showed a significant increase in number in a dose response manner (p < 0.05). This effect was observed at concentrations of 1.64, 3.28, 4.92, 6.56, 8.20 nM (48, 48, 50, 58 and 70%, respectively) (p < 0.05). Concentrations higher than 8.20 nM of CdS-MD nanoparticles also increased the number of viable cells; however, this effect decreased with the increase in concentration (60 to 20%) (p < 0.05). The morphological analysis of cells treated with 8.20 nM of CdS-MD nanoparticles revealed apoptosis in HepG2 cells, and a larger number of apoptotic cells were observed in breast cells (MDA-MB-231). On the other hand, we found minimal apoptotic cells but a considerable amount of division in the culture of intestinal cells (Figure 2B). It is interesting to note that 3% maltodextrin reduced the number of viable cells in all studied cell lines (12%) (p < 0.05).

### Characterization of cell death induced by CdS-MD nanoparticles in MDA-MB-231 cells

**Figure 3:**
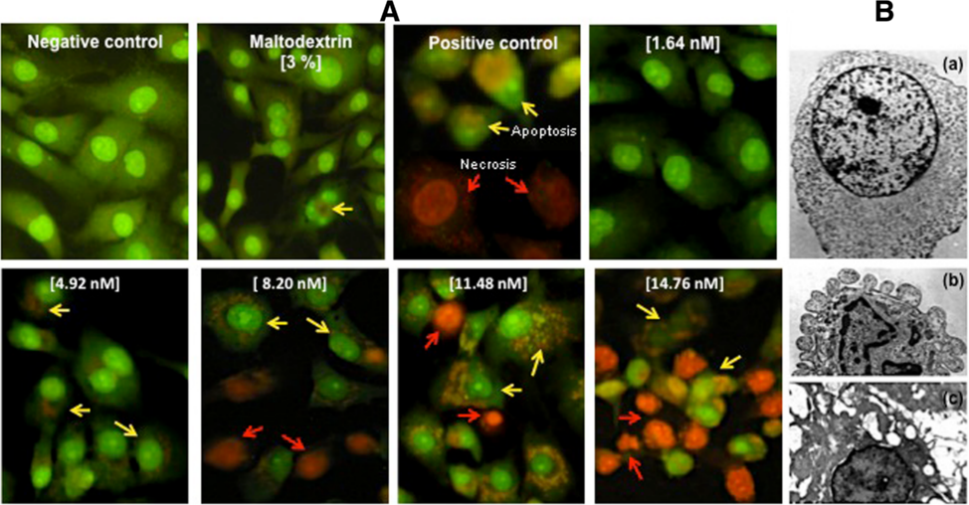
**Cell death induced by CdS-MD nanoparticles in MDA-MB-231 cells.** Cells were treated with CdS-MD nanoparticles (1.64-14.76 nM) for 24 h and stained with AO/EtBr staining and analyzed using fluorescence microscopy (100X). Cells exposed to 1μL/mL of 30% H_2_O_2_ for 2 h were used as apoptosis control and non-treated cells were used as negative control. These are representative results of at least three independent experiments (n = 3).

Because of MDA-MB-231 cells were the most sensitive to the cytotoxic effect of nanoparticles, we decided to make other assays in order to characterize the toxic effects of CdS-MD nanoparticles in this cell line. Acridin orange/ethidium bromide (AO/EtBr) double staining was used to differentiate between the apoptotic and necrotic cells. AO/EtBr staining revealed a dose (5% and 50% increase of apoptotic cells from 4.92-14.76 nM of CdS-MD nanoparticles) apoptotic cell death induction in MDA-MB-231 cells when exposed for 24 h (Figure 3A, yellow arrow). Ultra-structural analysis demonstrated that a significant portion of cells exposed to CdS-MD nanoparticles exhibit the morphological features of apoptosis (membrane blebbing, formation of apoptotic bodies and chromatin condensation) (Figure 3B). Cells exposed with 8.20, 11.48, 14.76 nM of CdS-MD nanoparticles also showed the presence of necrotic cells, which increased in a dose dependent manner (15%, 20% and 30%, respectively) (Figure 3A, red arrow). Maltodextrin treated cells showed scattered cells in apoptosis.

### Role of oxidative stress in apoptotic effects of CdS-MD nanoparticles

**Figure 4:**
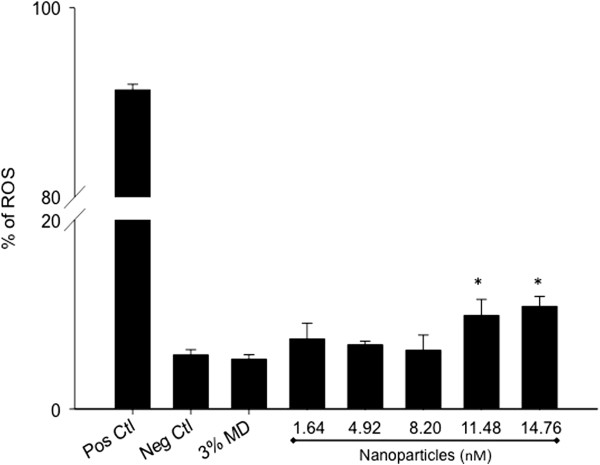
**Induction of oxidative stress in MDA-MB-231 cells by CdS-MD nanoparticles.** Cells were then exposed to CdS-MD nanoparticles (1.64-14.76 nM) for 2 h. Following treatment, cells were analyzed on a FacScalibur flow cytometer. 1μL/mL of 30% H_2_O_2_ served as a positive control for ROS induction in cells. Representative results of three independent experiments are presented as mean ± SD (n = 3). * Statistically different from control, p < 0.05.

Since oxidative stress has been given a putative role in apoptosis, we further evaluated the abilities of CdS-MD nanoparticles to cause oxidative stress in breast cells and studied the possible role of oxidative stress in apoptosis apoptotic induction by these CdS-MD nanoparticles. Figure 4 shows the quantitation of reactive oxygen species (ROS) production in cells after 2 hours exposure to CdS-MD nanoparticles. A significant increase in ROS production was observed in cells treated with 11.48 and 14.76 nM of CdS-MD nanoparticles (10-11%) (p < 0.05). No changes were observed with lower concentrations of CdS-MD nanoparticles or maltodextrin.

### Effect of CdS-MD nanoparticles on MDA-MB-231 cell proliferation

**Figure 5:**
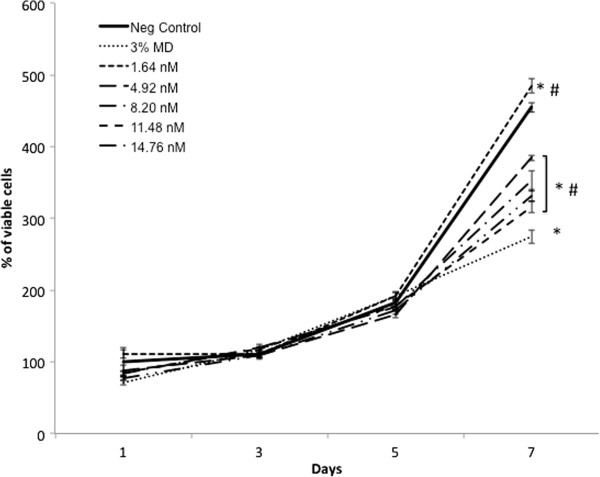
**Effect of CdS-MD nanoparticles on cell proliferation of MDAMB-231 cells.** Cells were followed for a period of 7 days after treatment with 1.64, 4.92, 8.20, 11.48 and 14.76 nM of CdS-MD nanoparticles. Data are presented as the mean ± SD of at least three independent experiments. * p < 0.05 as compared with control group. # p < 0.05 as compared with 3% maltodextrin group.

The analysis of cell proliferation in MDA-MB-231 showed different effects depending on the concentration; lower concentrations (1.64 nM of CdS-MD nanoparticles) led to an increase (6%) in cell proliferation at 7 days (p < 0.05). However, higher concentration (4.92, 8.20, 11.48 and 14.76 nM of CdS-MD nanoparticles) led to a significant reduction in cell proliferation in a dose response manner (15-30%) 7 days after treatment (Figure 5), p < 0.05. Cells treated with 3% maltodextrin affected MDA-MB-231 cell proliferation the most (40%) at 7 days. No significant changes were observed before 7 days.

### Evaluation of embryotoxicity

**Figure 6:**
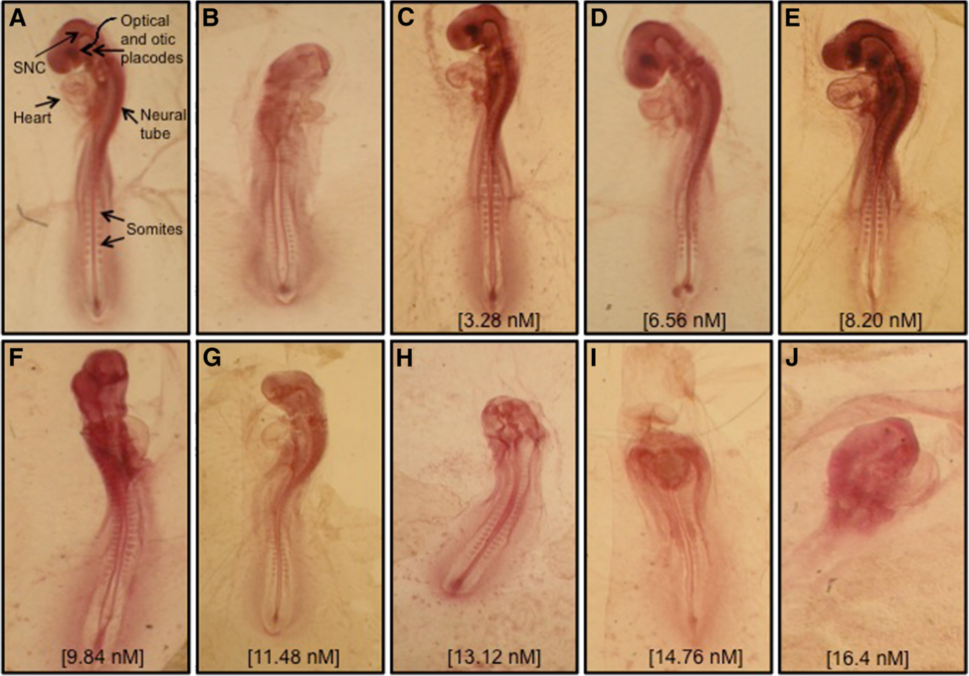
**Photographs of 72 h-old chick embryos. **(**A**) Non- treated embryo (control); (**B**) An embryo treated with 10 mg/mL caffeine (Positive control), which exhibited development defects; (**C-J**) Embryos of eggs treated with 3.28-16.4 nM of CdS-MD nanoparticles.

The present study evaluated the teratogenic effect of CdS-MD nanoparticles in a chicken embryo model. The results showed that treating chicken embryos with CdS-MD nanoparticles produced significant abortifacient activity. We found no significant changes in the weight, length or morphology of the embryos treated with concentrations lower than 8.20 nM of CdS-MD nanoparticles, but we did observe morphological alterations at concentrations higher than 9.84 nM of CdS-MD nanoparticles (Figure 6). The exposure of chicken embryos to CdS-MD nanoparticles resulted in a dose-dependent increase in anomalies that, starting at 9.84 nM, centered on the heart, central nervous system, placodes, neural tube and somites. No alterations were observed with concentrations of 3.28 nM of CdS-MD nanoparticles.

## Discussion

This study successfully synthesized maltodextrin coated cadmium sulfide semiconductor nanoparticles. The results show that CdS-MD nanoparticles can cause cytotoxicity, alter cell, proliferation and induce ROS production in human cell lines; however, the toxicity differed significantly depending on the cell type and CdS-MD nanoparticles concentration. We also showed evidence of the embryotoxic potential of CdS-MD nanoparticles when used in high concentrations. Although, quantum dots have attracted tremendous interest as luminiscent probes in biological and medical research due to their unique properties, their potential application in these fields has been limited due to their toxic effects [21,22]. Specifically, QDs contain toxic components such as cadmium [23]. Surface modification of QDs is therefore required to enhance stability.

The present study demonstrates that CdS-MD nanoparticles produced different effects on human cell lines, causing cytotoxic effects in MDA-MB-231 cells but inducing cell proliferation of HepG2 and Caco-2 cells depending on concentration. Indeed, some studies suggest that nanoparticles are not inherently benign and that they affect biological behavior at the cellular, subcellular, and protein levels [24-26]. Early studies by Kirchner et al. attempted to quantitatively determine values for the onset of cytotoxicity in CdSe and CdSe/ZnS quantum dots, either coated with mercaptopropionic acid (MPA), embedded in a silica shell or embedded in an amphiphilic polymer Shell [27]. They found that the majority of the nanoparticles were ingested into the cells and were stored in vesicles around the nucleus, irrespective of the surface coating. The toxic ions are commonly thought to be released from quantum dots when the surface of the nanoparticle is oxidized; early reports on the inclusion of simple quantum dots in bacteria support this [28]. Here we synthesized CdS nanoparticles coated with maltodextrin polymer and found cytotoxic effects at high concentrations. It is clear from this and other studies the surface coating is related to the toxicity experienced by the cells, which affects the level of toxic material released from the nanoparticles. The present study supports others indicating that different cell types have varying thresholds for quantum dots-induced toxicity.

Nanoparticles exposures can lead to disturbances in cellular homeostatic mechanisms, resulting either in adaptive cellular responses or cell death [29]. Cell death could occur either through an abrupt process named necrosis or by a tightly regulated or programmed process (apoptosis and autophagy) [13,30]. There has been a particular focus on DNA when looking at the effects of quantum dots in vitro given that DNA is known to be damaged by cadmium [31]. The morphological characterization of cell death in MDA-MB-231 cells was confirmed by immunofluorescence stain and by transmission electron microscopy (TEM) analysis of ultrastructure. TEM analysis confirmed the presence of the typical morphological features of apoptosis and necrosis in MDA-MB-231 cells after exposure to CdS-MD nanoparticles. Cells undergoing apoptosis show characteristic morphological features such as chromatin aggregation, nuclear and cytoplasmic condensation, partition of cytoplasm and nucleus into membrane bound-vesicles (apoptotic bodies); while the necrotic cells showed a loss membrane integrity, there was no vesicle formation and complete lysis. No cell deaths were observed when cells were treated with 1.64 nM of CdS-MD nanoparticles. However, apoptosis and necrosis were observed at concentrations higher 8.20 nM of CdS-MD nanoparticles, and this phenomenon increased in a dose dependent manner. The present report provides relatively consistent data on the cytotoxicity of QDs.

ROS play a dual role in cell fate, causing cell death as well as acting as second messengers to induce an adaptive cell response [32]. Oxidative stress has in fact been shown to induce cell death through a variety of mechanisms [30,33]. A hierarchical model for nanoparticles toxicity also describes the possibility of higher oxidative stress levels leading to cell death induction [34]. Different types of QDs have been shown to induce oxidative stress [35,36], and it was suggested that the photo-activation of QDs resulted in the generation of free radicals such as reactive oxygen intermediates (ROI), which would damage the DNA [37]. This study quantified the amount of ROS production in MDA-MB-231 cells. We found a significant increase in ROS production at concentrations of 11.48 and 14.76 nM of CdS-MD nanoparticles. It has been suggested that toxicity due to the production of reactive oxygen intermediates (ROI) is less controllable because it essentially has no barrier and occurs due to the resonance energy transfer from the quantum dots to molecular oxygen [38]. Lu et al. suggested that CdSe quantum dots were implicated in the apoptosis of human osteoblasts via the generation of ROI, causing the activation of certain enzymes that trigger apoptotic death [39]. Our results agree with this given that we found a significant rate of cell death at high CdS-MD nanoparticles concentrations.

QDs-induced perturbations of cellular mechanisms might act as a basis for different pathophysiological processes depending on concentration and the duration of exposure [21-23]. This study analyzed the effect of prolonged exposure to CdS-MD nanoparticles in MDA-MB-231 cells. We found that cells were alive after 24 hours and that cell proliferation during five days after exposition was not significantly affected; at 7 days, however, we found that cell proliferation had significantly increased with the lower concentration (1.64 nM) and was inhibited using concentrations higher than 4.92 nM at 7 days (p < 0.05). The results presented here indicate that although there initially was an adaptive response, the cytotoxic effect could not be completely eliminated. Proliferation changes in cells incubated with high concentrations suggest the presence of cell death or late cell arrest to repair the damage.

There are no published studies on QDs potential embryotoxicity in mammals. However, several *in vitro* and *in vivo* studies suggest local and systemic effects following exposure to nanoparticles [40]. Moreover, some nanoparticles readily travel throughout the body, deposit in target organs and get into many types of cells, lodge in mitochondria, and may trigger injurious responses [41]. Embryotoxicity is an important part of the toxicological profile of any new biologically active substance relevant to human safety. To reduce animal experimentation and predict *in vivo* embryotoxicity, *in vitro* tests like the chicken embryo model have been optimized [42]. Di Guglielmo et al. [43] used a zebrafish embryo model to demonstrate that gold and cobalt ferrite nanoparticles were able to modulate cell differentiation and induce weak embryotoxicity. Fein et al. [44] used the same model to demonstrate that fluorescent silica nanoparticles and/or aggregates mainly accumulate on the chorion of embryos and exhibit no overt embryotoxicity. By contrast, Bosman et al. [45] demonstrated that embryo development was not inhibited by exposure to polystyrene-based nanoparticles, suggesting a lack of embryotoxicity.

Our results showed that the tested CdS-MD nanoparticles were embryotoxic at high concentrations: a reduction in the axial skeleton and morphological changes in neural tube, somites, cardiovascular structure and central nervous system were observed. The embryotoxicity induced by cadmium was demonstrated early [46,47]. Present results indicate that the embryotoxicity mechanisms induced by CdS-MD nanoparticles have direct effects on developing tissue. The nature of the observed abnormalities suggests that these effects could be directly associated with concentration. However, embryotoxicity could also be explained by the chicken embryo model itself and the fact that the CdS-MD nanoparticles were added directly into the eggs.

## Conclusions

Our data indicates that CdS-MD nanoparticles have cytotoxic activity and may affect cell proliferation *in vitro*. They induce cell death by apoptosis and necrosis, which appear dependent on ROS production. They are also embryotoxic. However, the experimental results revealed that CdS-MD nanoparticles produced distinct dose-dependent effects. No toxic effects were observed with doses <3.28 nM. Therefore, the CdS-MD nanoparticles used in this study can be potentially used in bio-imaging applications. However, further studies using mammalian species are needed in order to discard more toxic effects.

## Methods

All chemicals were purchased from Sigma-Aldrich unless otherwise stated.

### Synthesis

Cadmium sulfide nanoparticles were prepared in aqueous solution. CdCl_2_ (5 mL, 0.02 M), KOH (10 mL, 0.5 M), NH_4_NO_3_ (5 mL, 0.5 M), CS(NH_2_)_2_ (5 mL, 0.2 M), were added and the mixture was stirred and heated at 80°C. Similar conditions were applied to maltodextrin with 3% concentration. These solutions were slowly added into the flask and adjusted to pH 10 using a dilute solution of sodium hydroxide. The solution immediately turned light yellow color indicating the initial formation of CdS nanoparticles. The temperature of the mixture was kept at 80°C and maintained at this temperature for 30 min. The yellow precipitate was isolated by centrifugation during 60 min at 6000 rpm. At the end of the process the yellow precipitate was washed several times with deionized water and acetone and finally dried at 40°C for 24h. In maltodextrin solution, the hydroxyl groups acted as stabilizer agents for the synthesized CdS nanoparticles. CdS nanoparticles have been synthesized using starch as capping agent [8].

### Nanoparticle characterization

The crystalline structure characterization of CdS-MD nanoparticles was done by powder X-ray diffraction (XRD) spectrometer (D5000, Siemens, Germany). CdS-MD nanoparticles were dispersed in ethanol and sonicated for 10 min and placed on a cupper-net for evaluation using a Jeol2010 TEM (Jeol, USA), transmission electron microscopy (TEM) images was used to determine the morphology and size of these nanoparticles. The emission spectrum of CdS-MD nanoparticles was carried out by luminescence spectrometer (LS55, Perkin Elmer, USA) using the excitation wavelength of 320 nm. The UV-visible spectrum of CdS-MD nanoparticles was recorded with a Perkin-Elmer Lambda 25 spectrophotometer.

### Cell culture

HepG2 (hepatocellular carcinoma), MDA-MB-231 (breast adenocarcinom), Caco-2 (colorectal adenocarcinoma) cell lines (ATCC, USA) were cultured in DMEM (GIBCO, USA), with 10% FBS (GIBCO, USA) and 100 U/ml penicillin/100 μg/ml streptomycin (GIBCO, USA), in a humidified 5% CO_2_ atmosphere at 37°C.

### Cell viability and cell proliferation assays

Cell viability and cell proliferation were determined using a MTT (methyl tetrazolium, Sigma Aldrich, USA) assay [48]. Briefly, for cell viability, HepG2, MDA-MB-231 and Caco-2 cells were seeded into a 96-well plate (10,000/well) and incubated for 24 h at 37°C and 5% CO_2_. The culture medium was replaced by a fresh one supplemented with different concentrations of CdS-MD nanoparticles (1.64, 3.28, 4.92, 6.56, 8.20, 9.84, 11.48, 13.12, 14.76 y 16.4 nM) and incubated for 24 h. For cell proliferation, HepG2, MDA-MB-231 and Caco-2 cells were seeded into a 96-well plate (1,000/well) and incubated for 24 h and then treated as described above for 1, 3, 5 and 7 days. After treatment, the medium was gently removed and replaced with 20 μL MTT (5 mg/mL) and 150 μL of non-phenol-red medium, and incubated for 4 h. Medium from each well was discarded, followed by the addition of 200 μL DMSO and 25 μL Sorensen’s glycine buffer (glycine 0.1 M, NaCl 0.1 M, pH 10.5) to each well. When the formazan crystals were dissolved, the optical density was determined on a microplate reader (Bio-Rad) at a wavelength 590 nm. Untreated cells served as a non-treatment control cell viability. The results represented a percentage of the relative viability of cells against to the untreated control. MTT results are presented as values relative to control values, expressed as percentages.

### Morphological analysis

Morphological analysis was performed by Giemsa (Sigma Aldrich, USA) staining of HepG2, MDA-MB-231 and Caco-2 cells. Cells were seeded into a 96-well plate (10,000/well) and incubated for 24 h at 37°C and 5% CO_2_. The medium was replaced with fresh medium containing 8.20 nM of CdS-MD nanoparticles. The plates were incubated for 24 h as described above. Cells were washed with DPBS and incubated with 1:2 DPBS-Methanol for 2 min at room temperature. Cells where then incubated with 100% methanol for 10 min and rinsed with DPBS. Images of stained cells were photographed with an Olympus digital camera. Morphological features of cell death induced by CdS-MD nanoparticles were studied using TEM. Fixation and Epon embedding of cells was performed as described elsewhere [49]. Ultrafine sections (60 nm thick) were collected on coppergrids and studied using a JEOL 1200 EXII microscope fitted with an energy dispersive spectrometer (OXFORDLINK ISIS 300).

### Assessment of cell death by fluorescence microscopy

The assessment of cell death was carried out using the acridine orange and ethidium bromide staining assay as described previously [50]. Briefly, the HepG2, MDA-MB-231 and Caco-2 cells were seeded into 6-well plate (250,000/well) and incubated for 24 h at 5% CO_2_ and 37°C. Culture medium was replaced with fresh media containing CdS-MD nanoparticles (1.64, 4.92, 8.20, 11.48 and 14.76 nM) and the cells were then incubated for another 24 h. After washing thoroughly with DPBS, 250 μL of a mixture of 100 μg/mL acridine orange/ 100 μg/mL ethidium bromide (Sigma Aldrich, USA) was added to the each well. The cells were then incubated at room temperature for 10 seconds and observed under a fluorescence microscope. Images of fluorescently stained cells were photographed with an Olympus digital camera. The data represents the average number of live, apoptotic or necrotic cells over at least 15 images for each treatment. Cells incubated in culture medium were used as a non-treated control. 1μL/mL of 30% H_2_O_2_ served as apoptosis control and smashed cells were used as necrosis control. Cells were categorized as healthy (green fluorescent cells without any nuclear staining), apoptotic (condensed or fragmented orange red nucleus) or necrotic (orange red, “apparently normal” or patchy nucleus).

### Reactive oxygen species (ROS) assay

The measurement of intracellular ROS levels was carried out using 2-7’-dichlorodihydrofluorescein diacetate (DCFH-DA) as described previously [51]. Briefly, HepG2, MDA-MB-231 and Caco-2 cells were seeded into 100 mm culture dishes (70-80% confluence) and incubated at 5% CO_2_ and 37°C. Cells were trypsinised and aliquoted into flow tubes at 300,000 cells per tube. Cells were then incubated with 5 μM DCFH-DA at 37°C and 5% CO_2_ for 30 min. The culture media containing CdS-MD nanoparticles (1.64, 4.92, 8.20, 11.48 and 14.76 nM) were added to the tubes in equal volume and incubated for 2 h under the incubation conditions described above. Following treatment, cells were centrifuged, resuspended in DPBS, and analyzed on a FacScalibur flow cytometer. 1 μL/mL of 30% H_2_O_2_ served as a positive control for the induction of intracellular ROS in cells.

### Embryotoxicity studies

A teratogenicity assay (chicken embryo assay) was carried out to determine the concentration dependency of CdS-MD nanoparticles teratogenicity, as described by Jelinek and Marthan [42]. Fertile White Leghorn chicken eggs were obtained from A.L.P.E. S.A. (Puebla, México) and stored at 6°C. A total of 100 fertilized eggs were weighed, sterilized, and divided into 10 groups. The first group served as a non-treated control. The next 8 groups received the CdS-MD nanoparticles treatment (3.28, 6.56, 8.20, 9.84, 11.48, 13.12, 14.76 and 16.4 nM). The last group received caffeine (10 mg/mL) and was considered positive control. Test solutions (1 mL) were added to the air sac under sterile conditions. Each solution was injected after drilling into the shell at the blunt end of the egg; after injection, the holes were immediately sealed with melted paraffin wax. The eggs were then transferred and maintained in a forced draft incubator at 37.5°C with a relative humidity of 55% until the desired stage of development was reached. Embryos in each group were fixed in buffered formal saline (pH 7.4), dehydrated, and embedded in paraffin blocks. Paraffin tissue sections of 6 μm were stained with acetocarmine for routine histological examination. The embryo was examined and staged according to the morphological criteria previously outlined by Hamburger and Hamilton [52]. Embryonic stages at the time of the CdS-MD nanoparticles application varied from 14 to 16, which approximately correspond to developed somites numbered 22 to 28.

### Statistical analysis

The data were represented as the mean ± SD of 3 independent experiments conducted by octuplicate. The data was statistically analyzed using the SPSS 10.0 software (SPSS Inc., Chicago, Ill., USA), the *t-test* and ANOVA. Differences were considered significant if the *P*-value was less than 0.05.

## Abbreviations

QD: Quantum dot; Cd^++^: Cadmio; CdS: Cadmiun sulfide; CdS-MD: Cadmium sulfide-maltodextrin; DE: Dextrose equivalent; XRD: X-ray diffractometer; TEM: Transmission electron microscopy; AO/EtBr: Acridine orange/ethidium bromide; ROS: Radical oxygen species; MPA: Mercaptopropionic acid; DNA: Dexoxy ribonucleic acid; ROI: Radical oxygen intermediates; EDS: Energy dispersive spectroscopy; DMEM: Dulbecco’s modified eagle medium; MTT: Tetrazolium salt.

## Competing interests

The authors declare that they have no competing interests.

## Authors’ contributions

PR synthesized and characterized the CdS-MD nanoparticles. AL carried out the cell culture assays. JR carried out the ROS analysis by flow cytometer and the morphological analysis. LR designed the study, performed the statistical analysis and wrote the paper. All authors red and approved the final manuscript.

## Authors’ information

PR Ph.D. researcher in Materials Science

AL Pharm. D. researcher in Pharmacy

JR M.D. and Ph.D., Professor and researcher in Physiology

LR M.D. and Ph.D., Professor and researcher in Pharmacology and Toxicology.
